# Predictors for High-Risk Carotid Plaque in Asymptomatic Korean Population

**DOI:** 10.1155/2020/6617506

**Published:** 2020-12-30

**Authors:** Chae Won Jang, Yong Kyun Kim, Ki-Hong Kim, Achangwa Chiara, Moo-Sik Lee, Jang-Ho Bae

**Affiliations:** Cardiovascular Center, Division of Cardiology, Department of Internal Medicine, Konyang University Hospital, Daejeon, Republic of Korea

## Abstract

**Aims:**

High-risk carotid plaque remains an important risk factor for atherosclerotic cardiovascular disease (ASCVD). We sought to evaluate the characteristics of carotid plaque and to find out the predictors for high-risk carotid plaque in asymptomatic Koreans.

**Methods:**

Subjects (*n* = 801) without a history of ASCVD from 12 university hospitals in Korea underwent carotid ultrasound. The images were standardized at core laboratory. Morphologic characteristics of plaque were analyzed with laboratory and clinical characteristics. High-risk carotid plaque features included the highest quartile of carotid plaque score (cPS), irregular plaque surface, and hypoechoic and ulcerated plaque.

**Results:**

The carotid plaque prevalence was 22.1% (177/801 persons, 293 plaques). The plaque was increased with age (*p* < 0.001) and conventional ASCVD risk estimator (*p* < 0.001) and the most frequently found in bulb (*n* = 190, 64.8%). The number of the highest quartile of cPS was 44/177 (24.9%). Irregular plaque was seen in 20.8% out of total plaque (61/293) and was more frequent in the high-risk 10-year ASCVD risk group than in the low-risk group (36.1% vs. 15.8%, *p* = 0.023). Hypoechoic and ulcerated plaques were seen in 14.3% (42/293) and 2% (6/293), respectively. The independent predictors for high-risk plaque were age (*β* = 0.052, *p* < 0.001), HbA1c (*β* = 0.182, *p* = 0.004), male (*β* = 0.118, *p* = 0.006), hypertension (*β* = 0.090, *p* = 0.032), and multiple plaques (OR: 4.810 (two plaques) and 8.621 (three plaques), all *p* < 0.001).

**Conclusions:**

This study suggests that high-risk carotid plaque was seen in 12.4% (99/801). The high-risk plaque was associated with diabetes control status reflected by the HbA1c level as well as traditional risk factors in asymptomatic Korean population.

## 1. Introduction

Atherosclerotic cardiovascular disease (ASCVD) is a leading cause of morbidity and mortality in the world. According to the national statistics of Korea in 2018 [1], heart disease was the second cause of death (62.4 persons/100,000) increasing from third cause (43.4 persons/100,000) in 2008. Cerebrovascular disease is the fourth cause of death (44.7 persons/100,000) in 2018.

Routine measurement of carotid intima-media thickness (CIMT) is no longer recommended to assess atherosclerosis. However, carotid plaque burden and thickness are reported as a surrogate of atherosclerosis and can predict future ASCVD [2, 3].

Epidemiologic and clinical importance of plaque was investigated in many studies [4, 5]. The Atherosclerosis Risk in Communities (ARIC) study [5] showed that 33.6% of carotid plaque prevalence and whites (34.4%) had higher prevalence than blacks (31.4%). Caucasians had more plaque formation and higher plaque score than African-American, Hispanic, and Chinese over a decade in the Multi-Ethnic Study of Atherosclerosis (MESA) study [4]. Free-living Koreans in their 35 to 64 years had carotid plaque (17%) [6]. Carotid plaque was associated with cardiovascular events in both healthy population and patients with coronary artery disease [7–10].

Specific morphological features of carotid plaque such as echogenicity, ulcerated, or irregular surface are an important parameter to predict major adverse cardiovascular event (MACE) [11–15]. Nevertheless, prevalence, morphologic characteristics, and predictors for high-risk carotid plaque have not been studied in a large number of study population, especially in Asian.

This study is aimed at evaluating morphologic characteristics of carotid plaque and seeking the predictors of high-risk carotid plaque in a large number of asymptomatic Koreans.

## 2. Patients and Methods

### 2.1. Study Population

This was an observational cohort study. We had already reported the characteristics of CIMT in the previous study with this cohort [16]. Subjects who were not referred for ASCVD or symptoms were enrolled in the study, and informed consent was provided. A total of 1,957 participants were enrolled between May 2010 and August 2013 from 12 university hospitals in Korea. We excluded 131 subjects from this study: coronary artery disease (*n* = 100), cerebrovascular disease (*n* = 29), and end-stage renal disease (*n* = 2). Additional 1,025 subjects were excluded to calculate 10-year ASCVD risk with the pooled cohort equation (PCE) [17]. Detailedly, there were 726 subjects having incomplete data, 185 subjects who were <40 or >79 years old, 111 subjects who had total cholesterol level < 130 mg/dL or >320 mg/dL, and 3 subjects with systolic pressure higher than 200 mmHg according to the exclusion criteria of the estimator. Finally, the study population consisted of 801 subjects who had whole carotid artery images, gave informed consent and proper cholesterol and blood pressure range for the equation, and aged 40-79 years without ASCVD history.

This study was approved by the ethics committee of Konyang University Hospital and conducted in accordance with the Declaration of Helsinki.

### 2.2. Carotid Artery Imaging

Carotid artery scanning was fully performed according to the guideline suggested by the American Society of Echocardiography [18]. The carotid artery was examined by a high-resolution ultrasonographic unit with a linear array transducer (set to 11 MHz). The subjects were assessed in a supine position with mild head extension. The depth control was fixed at 4 to optimize the image quality. The clear images were captured digitally and sent to the Cardio-Cerebrovascular Data Center in Korea Research Institute of Standards and Science for later off-line core laboratory analysis. We defined carotid plaque as focal structures protruding into the arterial lumen more than 0.5 mm or 50% of the surrounding IMT or its CIMT ≥ 1.5 mm [19].

Carotid plaque was assessed by two competent professionals (CWJ and KHK) regarding site, number, width, thickness, stenosis, surface irregularity, ulcer, and echogenicity. The senior professional (JHB) did the final report about the characteristics of the plaque, if the two professionals disagreed with each other's description. The analysis was performed in the common carotid artery, carotid bulb, and internal and external carotid artery of both sides. Size and stenosis were manually measured using electronic calipers. Carotid plaque width was measured from proximal to distal junction between the normal intima and plaque in the longitudinal view showing the largest extent of the plaque. Carotid plaque thickness was measured on the thickest part of the plaque in the longitudinal view. Irregular plaque was defined as the plaque depth variation between 0.4 and 2 mm along the contour of the lesion [20]. Ulcerated plaque was defined as the plaque with a focal defect which is at least 2 mm deep with a well-defined back wall at its base and with revered flow in the color Doppler [20]. Echogenicity of carotid plaque was classified into 3 groups: hypoechoic, isoechoic, and hyperechoic plaques. We used 2 reference echo structures (vessel lumen and adventitia). Hypoechoic plaque was defined as plaque echogenicity similar with vessel lumen. It appeared black or almost black. Hyperechoic plaque was defined as plaque echogenicity with the adjacent adventitia, used as a reference structure. Isoechoic plaque was defined as which appeared echogenicity between hypoechoic and hyperechoic plaques [21]. Carotid stenosis was calculated by the European Carotid Surgical Trial (ECST) using obtained longitudinal B-mode images showing the thickest plaque. The formula was %carotid stenosis = (1 − [residual diameter of the shortest lumen/original diameter of the diseased artery]) × 100 [22]. Arterial diameter was measured from the near wall media and adventitia junction to far wall media and adventitia junction [23]. Carotid plaque score (cPS) was defined as the sum of maximal thickness of the plaques located in the common carotid artery, carotid bulb, and internal carotid artery of both sides [24]. High-risk plaque was defined as carotid plaque with at least one of the following morphologic features: hypoechoic plaque, irregular plaque, ulcerated plaque (supplementary figure 1), and the highest 25% of cPS.

### 2.3. Clinical Characteristics and Laboratory Data

Clinical data included age, sex, medical history, medication, smoking behavior (current smoker or not), alcohol consumption, weight, height, body mass index (BMI), and blood pressure. Age was divided into 6 groups according to Systemic Coronary Risk Estimation (SCORE) [25]: 40-49, 50-54, 55-59, 60-64, 65-69, and ≥70 years of age. The status of weight was classified into 4 groups by BMI (kg/m^2^): underweight (<18.5), normal (18.5-24.9), obese I (25-29.9), and obese II (≥30). Medical history included hypertension (blood pressure ≥ 140/90 mmHg based on the average of three times repeated readings or patients on antihypertensive drugs), diabetes (controlled with diet, oral hypoglycemic agents, or insulin, fasting glucose level ≥ 126 mg/dL, or HbA1c ≥ 6.5%), stage 3-5 chronic kidney disease (eGFR < 60 mL/min/1.73m^2^), and dyslipidemia. Dyslipidemia was categorized into 5 groups using criteria based on the National Cholesterol Education Program/Adult Treatment Panel-III (NCEP-ATP III) guidelines [26] that define low-density lipoprotein-cholesterol (LDL-C), high-density lipoprotein-cholesterol (HDL-C), and triglyceride thresholds as abnormal: combined hyperlipidemia, hypercholesterolemia, metabolic syndrome, hypertriglyceridemia, and low HDL-C.

Obtained laboratory data was fasting glucose, HbA1c, lipid profile (total cholesterol, LDL-C, HDL-C, and triglyceride), BUN, creatinine, and high-sensitivity CRP, which was collected within 2 weeks of enrollment.

Framingham risk score (FRS) and PCE were used to investigate the cardiovascular risk. FRS consisted of low- (<10%), intermediate- (≥10 to ≤20%), and high- (>20%) risk groups [27]. PCE was classified for 3 groups: low (<5%), borderline/intermediate (≥5 to <20%), and high (≥20%), in this study [28].

### 2.4. Statistical Analysis

All data were analyzed using SPSS version 18.0 (SPSS, Inc., Chicago, IL, USA). A *p* value of 0.05 was considered statistically significant. Variables were expressed as absolute number, percentages, and mean ± standard deviation. Comparisons of group which had carotid plaque or not were performed by the Student *t*-test and chi-square test. The difference between 10-year ASCVD risk and plaque prevalence was analyzed by the extended Mantel-Haenszel method of chi-square for linear trend. The predictors for carotid plaque, irregular plaque, and hypoechoic plaque were evaluated by multivariate logistic regression analysis, and the highest quartile cPS was analyzed by multiple regression analysis.

## 3. Results

### 3.1. Demographics

The mean age was 57.9 ± 10.3 years old, and there were 403 men (50.3%) in a total of 801 study subjects (Table 1). Plaque was present in 177 subjects (22.1%). The group with carotid plaque had more male, older, higher prevalence of hypertension, diabetes, higher levels of fasting glucose, HbA1, total cholesterol, LDL-C, and statin than those without plaque. There were no other differences in demographics between the two groups.

### 3.2. Carotid Sonographic Plaque Findings

A total number of 293 carotid plaques were found in 177 subjects (Table 2). Ninety-two subjects (52%) had a single plaque, and 85 subjects (48%) had multiple plaques. The mean number of plaques was 1.66 ± 0.76, and cPS was 3.67 ± 1.96. The thickness and width of plaque were 2.25 ± 0.69 mm and 12.04 ± 6.10 mm, respectively. The mean stenosis of carotid artery was 30.51 ± 12.67%.

The plaque was most frequently found in bulb (64.8%), followed by common carotid artery (24.6%), internal carotid artery (8.9%), and external carotid artery (1.7%). There was no difference between the right (*n* = 146) and the left (*n* = 147) sides of carotid artery.

### 3.3. Characteristics and Predictors of High-Risk Plaque

High-risk carotid plaque features included the highest quartile of cPS, irregular plaque surface, hypoechoic plaque, and ulcerated plaque.

The highest quartile of cPS was >4.82. Age (*β* = 0.255, *p* < 0.001) was the strongest predictor of cPS in a model of multivariate linear regression (Table 3). HbA1c (*β* = 0.121, *p* = 0.004), male (*β* = 0.118, *p* = 0.006), and hypertension (*β* = 0.090, *p* = 0.032) were also significantly associated with cPS. The increased level of HbA1c was an important predictor not only for all subjects but also for diabetes patients (*β* = 0.170, *p* = 0.004).

Plaques with irregular surfaces were observed in 61 plaques of 52 participants. Irregular plaque was more frequent in the high-risk PCE group than in the low-risk PCE group (36.1% vs. 15.8%, *p* = 0.023). Multiple plaques were a risk factor of irregular plaque. The odds ratio (OR) of irregular plaque was 4.810 for subjects with two plaques, and OR was 8.621 for those with three plaques (all *p* < 0.001).

Thirty-nine participants had hypoechoic plaque (42 plaques). Hypoechoic plaque was inversely associated with aging (≥70 years, OR 0.186, 95% confidence interval (CI) 0.050-0.692, *p* = 0.012). However, advanced age was a risk factor for hyperechoic plaque (OR 1.044, 95% CI 1.005-1.084, *p* = 0.027).

Six participants had ulcerated plaque (6 plaques). Plaque ulcer was not associated with ASCVD risk stratification.

### 3.4. Predictors of Carotid Plaque

The univariate logistic regression analysis showed that the important predictors of carotid plaque were male (OR 1.503, 95% CI 1.073-2.107, *p* = 0.018), age (OR 1.071, 95% CI 1.053-1.090, *p* < 0.001), hypertension (OR 1.997, 95% CI 1.416-2.817, *p* < 0.001), diabetes (OR 2.077, 95% CI 1.481-2.911, *p* < 0.001), fasting glucose (OR 1.006, 95% CI 1.002-1.009, *p* = 0.001), HbA1c (OR 1.261, 95% CI 1.100-1.447, *p* = 0.001), LDL-C (OR 0.991, 95% CI 0.985-0.997, *p* = 0.002), FRS (intermediate: OR 2.067, 95% CI 1.353-3.157, *p* = 0.001; high: OR 3.030, 95% CI 1.715-5.353, *p* < 0.001), and PCE (borderline/intermediate: OR 2.028, 95% CI 1.331-3.092, *p* = 0.001; high: OR 4.766, 95% CI 2.965-7.662, *p* < 0.001).

Multivariate logistic regression analysis (Table 4) showed that the independent predictors for carotid plaque presence were male (OR 1.637, 95% CI 1.131-2.368, *p* = 0.009), ≥60 years old (60-64 yrs: OR 2.014, 95% CI 1.029-3.943, *p* = 0.041; 65-69 yrs: OR 4.043, 95% CI 2.081-7.855, *p* < 0.001; ≥70 yrs: OR 6.775, 95% CI 3.717-12.349, *p* < 0.001), hypertension (OR 1.625, 95% CI 1.125-2.347, *p* = 0.010), and diabetes (OR 1.590, 95% CI 1.104-2.290, *p* = 0.013).

### 3.5. Carotid Plaque and Traditional ASCVD Risk

**Figure 1:**
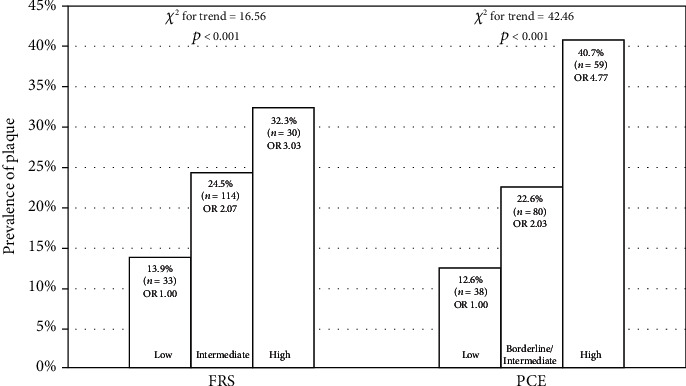
Association between plaque prevalence and ASCVD risk stratification (FRS and PCE). There was a significant linear trend among the ordered categories defining the 10-year ASCVD risk (FRS and PCE) and the prevalence of plaques. ASCVD: atherosclerotic cardiovascular disease; FRS: Framingham risk score; PCE: pooled cohort equation; OR: odds ratio.

The prevalence of plaque was significantly increased with higher ASCVD risk stratification of both FRS and PCE (Figure 1). Carotid plaque prevalence was 13.6% in the low FRS risk group, 24.5% in the intermediate-risk group, and 32.3% in the high-risk group. When calculated in PCE, the prevalence of plaque was 12.6% in low risk, 22.6% in borderline/intermediate risk, and 40.7% in high risk.

In addition to the prevalence of carotid plaque, there was a difference in plaque number and score between each ASCVD risk (supplementary table 1). Particularly, the cPS significantly increased, as the FRS and PCE risk group increased.

## 4. Discussion

This study is the first to show the prevalence and predictors of high-risk carotid plaque in asymptomatic Korean population to the best of our knowledge.

The main finding of this study was the prevalence and predictors of high-risk carotid plaque in asymptomatic Koreans. The prevalence of high-risk plaques was 42/293 (14.3%) with hypoechoic plaque, 61/293 (20.8%) with irregular plaque, 6/293 (2%) with ulcerated plaque, and 44/177 (24.9%) with high cPS. The independent predictors for high-risk plaque were age, HbA1c, male, hypertension, and multiple plaques.

The interesting finding was that HbA1c was an important predictor of high-risk plaque. This finding suggests that diabetes control status may be important in high-risk carotid plaque feature, although diabetes itself was an important factor for carotid plaque presence, which was shown in many clinical studies as well as our study. Our previous study [29] showed that well-controlled diabetic patients (HbA1c < 7%) had similar coronary plaque composition and plaque volume with nondiabetic patients, whereas poorly controlled diabetes had larger dense calcium volume and necrotic core volume. It is consistent with the result of this current study in regard to atherosclerosis. That is, poorly controlled diabetes is associated with unfavorable findings of coronary or carotid plaque.

The elevated level of LDL-C has an important role in the pathogenesis of atherosclerosis. However, our data showed that the level of LDL-C was lower in a group with plaque (104.7 ± 29.2 mg/dL) than in those without plaque (112.8 ± 30.1 mg/dL). It could be explained by the result that subjects with carotid plaque (*n* = 72, 55%) took statin more than those without plaque (*n* = 107, 25.4%) significantly (*p* < 0.001).

Additionally, smoking is a risk factor for the development of cardiovascular disease. Current smokers had strong association with the presence of carotid plaque [30]. In our study, smoking status did not differ between the two groups. However, cPS was associated with current smoking in the <50-year-old group (*β* = 0.151, *p* = 0.038), whereas older did not. The result suggests that smoking cessation is a strong modifiable risk factor to preventing ASCVD, especially among younger individuals. Further study is needed to determine the relationship between plaque and duration or level of smoking.

The ARIC study [5] showed 33.6% prevalence of plaques and whites (34.4%) had more plaque than blacks (31.4%). The MESA study [31] found that the prevalence of carotid plaque was 43.5%. German males aged 45 to 54, without cardiovascular disease, had 27.8% of prevalence of plaque [32]. Healthy whites (22.8%) with a mean age of 45 years had a higher carotid plaque burden than Japanese (4.8%) and Koreans (10.6%) [33]. The prevalence of carotid plaque for Chinese between 30 and 79 years old was 20.15% [34]. The above studies using similar carotid plaque definition (focal IMT ≥ 1.5 mm) with our study showed that there was a clear racial difference indicating Western people (22.8%~43.5%) had higher prevalence of carotid plaque than Asian people (4.8%~22.1%).

Carotid arterial plaque burden, not CIMT, is still regarded as class IIa in risk stratification for cardiovascular disease by 2019 ESC/EAS guidelines [25]. Furthermore, some morphologic features of plaque (hypoechoic, irregular, and ulcerated plaques and high cPS) are associated with poor clinical outcomes such as increased transient ischemic attack, stroke, myocardial infarction, rehospitalization for a cardiovascular-related illness, and all causes of death [11, 13, 15, 35]. Out of the high-risk plaque features, cPS as well as carotid plaque burden could predict the event rate of ASCVD in asymptomatic Americans [2]. Therefore, we measured and used cPS as one of the high-risk plaque features. To the best of our knowledge, the study showing predictors for high-risk carotid plaque features has not been reported up to now. Our study suggested that the predictors for high-risk carotid plaque were age, HbA1c, male, hypertension, and multiple plaques. Although hypoechoic plaque is one of the high-risk plaques, our study showed that aging was inversely associated with hypoechoic plaque. Hypoechoic plaque consists of lipid, thrombi, and hemorrhages. It is associated with complex coronary plaque and was an indicator for future coronary events [36, 37] and multiplies the risk of stroke by 2.31 times [11]. However, hyperechoic plaque with diffuse calcification is associated with stable or healed ruptured plaque and statin therapy which can enhance calcification in the necrotic core, whereas spotty calcification is generally associated with unstable plaques [38]. Diffuse calcification is increased with aging. These pathological processes could explain why hypoechoic plaque was less frequently found in old age and hyperechoic plaque showed a positive relationship with age in our study. This result suggests that the examiner should perform carotid ultrasonography carefully to identify hypoechoic plaque especially for the younger group and to distinguish diffuse calcification from spotty calcification for hyperechoic plaque in the older group.

### 4.1. Limitations

Our study has several limitations. Firstly, this was a retrospective study and could not include all subjects who underwent carotid ultrasonography because of lack of data. All subjects without ASCVD (*n* = 1,826, mean age 57.4 ± 13.9 years old) had 20.6% prevalence of plaque. The result was not significantly affected by this loss of subjects, although our report showed higher prevalence (22.1%, mean age 57.9 ± 10.3 years old). Secondly, we have not known the prospective relationship between high-risk plaque and future atherosclerotic events because our study design did not account for it. This is a clinically important issue as currently we are carrying our study with MACE selected as primary end-point according to carotid plaque status. Finally, the data had limitation to distinguish the type, duration, and dose of lipid-lowering agents. Further studies are needed to evaluate the impact of medications on plaque.

## 5. Conclusions

The prevalence of high-risk carotid plaque in asymptomatic Koreans was 12.4%. The important predictors for high-risk carotid plaque (the highest quartile of cPS, irregular surface, and hypoechoic and ulcerated plaque) were age, HbA1c, male, hypertension, and multiple plaques. This study suggests that diabetes control status reflected by the HbA1c level was associated with high-risk features of carotid plaque in asymptomatic Koreans.

## Figures and Tables

**Table 1:** Demographics of the subjects according to the presence of plaque.

	Total (*n* = 801)	Plaque (+) (*n* = 177, 22.1%)	Plaque (-) (*n* = 624, 77.9%)	*p* value
Male, *n* (%)	403 (50.3)	103 (58.2)	300 (48.1)	0.018
Age (years)	57.9 ± 10.3	63.3 ± 10.1	56.3 ± 9.8	<0.001
Age group, *n* (%)				<0.001
40-49 years	188 (23.6)	19 (10.1)	169 (89.9)	
50-54 years	150 (18.7)	25 (16.7)	125 (83.3)	
55-59 years	123 (15.4)	19 (15.6)	103 (84.4)	
60-64 years	111 (13.9)	24 (21.6)	87 (78.4)	
65-69 years	90 (11.2)	29 (32.2)	61 (67.8)	
≥70 years	138 (17.2)	61 (44.2)	77 (55.8)	
Systolic blood pressure (mmHg)	123.5 ± 17.2	124.1 ± 18.9	123.3 ± 16.6	0.637
Diastolic blood pressure (mmHg)	76.6 ± 11.9	75.4 ± 11.8	16.9 ± 11.9	0.132
BMI (kg/m^2^)	24.2 ± 3.0	24.3 ± 3.1	24.2 ± 3.0	0.677
Obesity (BMI)				0.836
Underweight (<18.5 kg/m^2^), *n* (%)	13 (1.7)	2 (15.4)	11 (84.6)	
Normal (18.5-24.9 kg/m^2^), *n* (%)	411 (52.8)	86 (20.9)	325 (79.1)	
Obese I (25-29.9 kg/m^2^), *n* (%)	317 (40.7)	73 (23.0)	244 (77.0)	
Obese II (≥30 kg/m^2^), *n* (%)	38 (4.9)	9 (23.7)	29 (76.3)	
Smoking, *n* (%)	166 (20.7)	39 (22)	127 (20.4)	0.626
Hypertension, *n* (%)	401 (50.1)	112 (63.3)	289 (46.3)	<0.001
Diabetes, *n* (%)	310 (38.7)	93 (52.5)	218 (34.9)	<0.001
CKD, *n* (%)	53 (7.0)	17 (9.9)	36 (6.1)	0.087
Fasting glucose (mg/dL)	119.8 ± 43.8	129.7 ± 48.5	116.9 ± 41.9	0.002
HbA1c (%)	6.6 ± 1.3	6.9 ± 1.5	6.5 ± 1.3	0.002
Lipid combination				0.500
Normolipidemia, *n* (%)	364 (45.7)	85 (48.0)	279 (45.1)	
Combined hyperlipidemia, *n* (%)	17 (2.1)	6 (3.4)	11 (1.8)	
Hypercholesterolemia, *n* (%)	32 (4.0)	4 (2.3)	28 (4.5)	
Metabolic syndrome, *n* (%)	105 (13.2)	20 (11.3)	85 (13.7)	
Low HDL cholesterolemia, *n* (%)	160 (20.1)	35 (19.8)	125 (20.2)	
Hypertriglyceridemia, *n* (%)	118 (14.8)	27 (15.3)	91 (14.7)	
Total cholesterol (mg/dL)	185.3 ± 33.6	176.9 ± 32.2	187.7 ± 33.7	<0.001
Triglyceride (mg/dL)	139.9 ± 122.8	134.0 ± 88.9	141.6 ± 118.7	0.425
HDL, cholesterol (mg/dL)	51.2 ± 12.6	50.8 ± 12.7	51.3 ± 12.6	0.648
LDL, cholesterol (mg/dL)	111.0 ± 30.1	104.7 ± 29.2	112.8 ± 30.1	0.002
Statin, *n* (%)	179/553 (32.4)	72/131 (55)	107/422 (25.4)	<0.001

Continuous variables are shown as mean ± standard deviation, and categorical variables are shown as absolute number and proportions. BMI: body mass index; CKD: chronic kidney disease (eGFR < 60 mL/min/1.73 m^2^); HDL: high-density lipoprotein; LDL: low-density lipoprotein.

**Table 2:** Characteristics of subjects and carotid plaque.

Characteristics of subjects with plaque (*n*)	177
Mean number of plaques, *n*	1.66 ± 0.76
Carotid plaque score	3.67 ± 1.96
Single plaque, *n* (%)	92 (52)
Multiple plaques, *n* (%)	85 (48)
Two plaques, *n* (%)	54 (30.5)
Three plaques, *n* (%)	31 (17.5)

Characteristics of plaque (*n*)	293
Site	
Common carotid artery, *n* (%)	72 (24.6)
Right/left (*n*)	36/36
Carotid bulb, *n* (%)	190 (64.8)
Right/left (*n*)	92/98
Internal carotid artery, *n* (%)	26 (8.9)
Right/left (*n*)	15/11
External carotid artery, *n* (%)	5 (1.7)
Right/left (*n*)	3/2
Size	
Thickness (mm)	2.25 ± 0.69
Width (mm)	12.04 ± 6.10
Stenosis (%)	30.5 ± 12.7
Echogenicity	
Hypoechoic, *n* (%)	42 (14.3)
Isoechoic, *n* (%)	203 (69.3)
Hyperechoic, *n* (%)	48 (16.4)
Ulceration, *n* (%)	6 (2.0)
Irregularity, *n* (%)	61 (20.8)

Continuous variables are shown as mean ± standard deviation, and categorical variables are shown as absolute number and proportions.

**Table 3:** Predictors of high-risk plaque.

	*B* (95% CI of *B*)	*β*	*p* value
A. The highest quartile of cPS			
Age (years)	0.052 (0.035-0.069)	0.255	<0.001
HbA1c (%)	0.182 (0.058-0.306)	0.121	0.004
Gender (male = 1, female = 0)	0.477 (0.138-0.816)	0.118	0.006
Hypertension (mmHg)	0.369 (0.031-0.707)	0.090	0.032

A-1. For diabetic patients			
Age (years)	0.054 (0.028-0.081)	0.239	<0.001
HbA1c (%)	0.265 (0.087-0.443)	0.170	0.004
Hypertension (mmHg)	0.683 (0.179-1.188)	0.154	0.008

	OR	95% CI of OR	*p* value
B. Irregular plaque			
Two plaques (Ref. single plaque)	4.810	2.029-11.402	<0.001
Three plaques	8.621	3.272-22.716	<0.001

C. Hypoechoic plaque			
≥70 years (Ref. 40-49 years)	0.186	0.050-0.692	0.012

(A) Multiple regression analysis. The predictors of the highest quartile of cPS. (A-1) The predictors of the highest quartile of cPS for diabetic patients. Higher HbA1c was a strong predictor. (B, C) Multivariate logistic regression analysis. cPS: carotid plaque score; HbA1c: glycated hemoglobin; *B*: unstandardized coefficient; *β*: standardized beta; OR: odds ratio; CI: confidence interval.

**Table 4:** Predictors of carotid plaques.

Variables	OR	95% CI of OR	*p* value
Male (Ref. female)	1.637	1.131-2.368	0.009
Age (Ref. 40-49 years)			
50-54 years	1.689	0.883-3.232	0.113
55-59 years	1.437	0.718-2.873	0.306
60-64 years	2.014	1.029-3.943	0.041
65-69 years	4.043	2.081-7.855	<0.001
≥70 years	6.775	3.717-12.349	<0.001
Hypertension (Ref. normotension)	1.625	1.125-2.347	0.010
Diabetes (Ref. nondiabetes)	1.590	1.104-2.290	0.013

Logistic regression analysis. Ref.: reference; OR: odds ratio; CI: confidence interval.

## Data Availability

It is impossible to access data from outside to protect the privacy of research subjects and the confidentiality of their personal information.

## Supplementary Materials

Supplementary Materials Supplementary Figure 1: high-risk carotid plaque. Supplementary Table 1: the difference of carotid plaque number and score according to ASCVD risk.
