# Supplementary material for: Predictors for High-Risk Carotid Plaque in Asymptomatic Korean Population
Source: Cardiovasc Ther. 2020 Dec 30;2020:6617506. doi: 10.1155/2020/6617506 (PMC7787813; doi:10.1155/2020/6617506)
Supplement: Supplementary Materials — Supplementary Figure 1: high-risk carotid plaque. Supplementary Table 1: the difference of carotid plaque number and score according to ASCVD risk. [file 6617506.f1.docx]

**Supplementary figure 1.** High risk carotid plaque



| 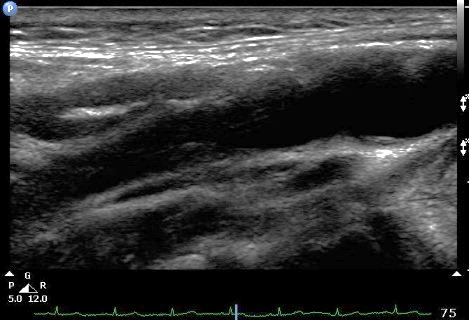  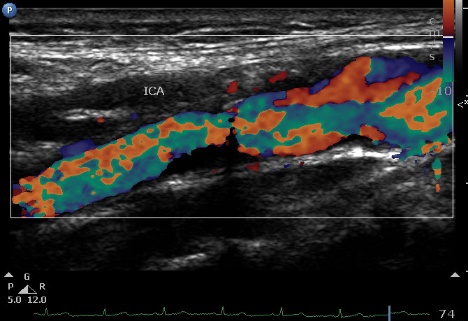 | (A) Hypoechoic plaque |
| --- | --- |
| 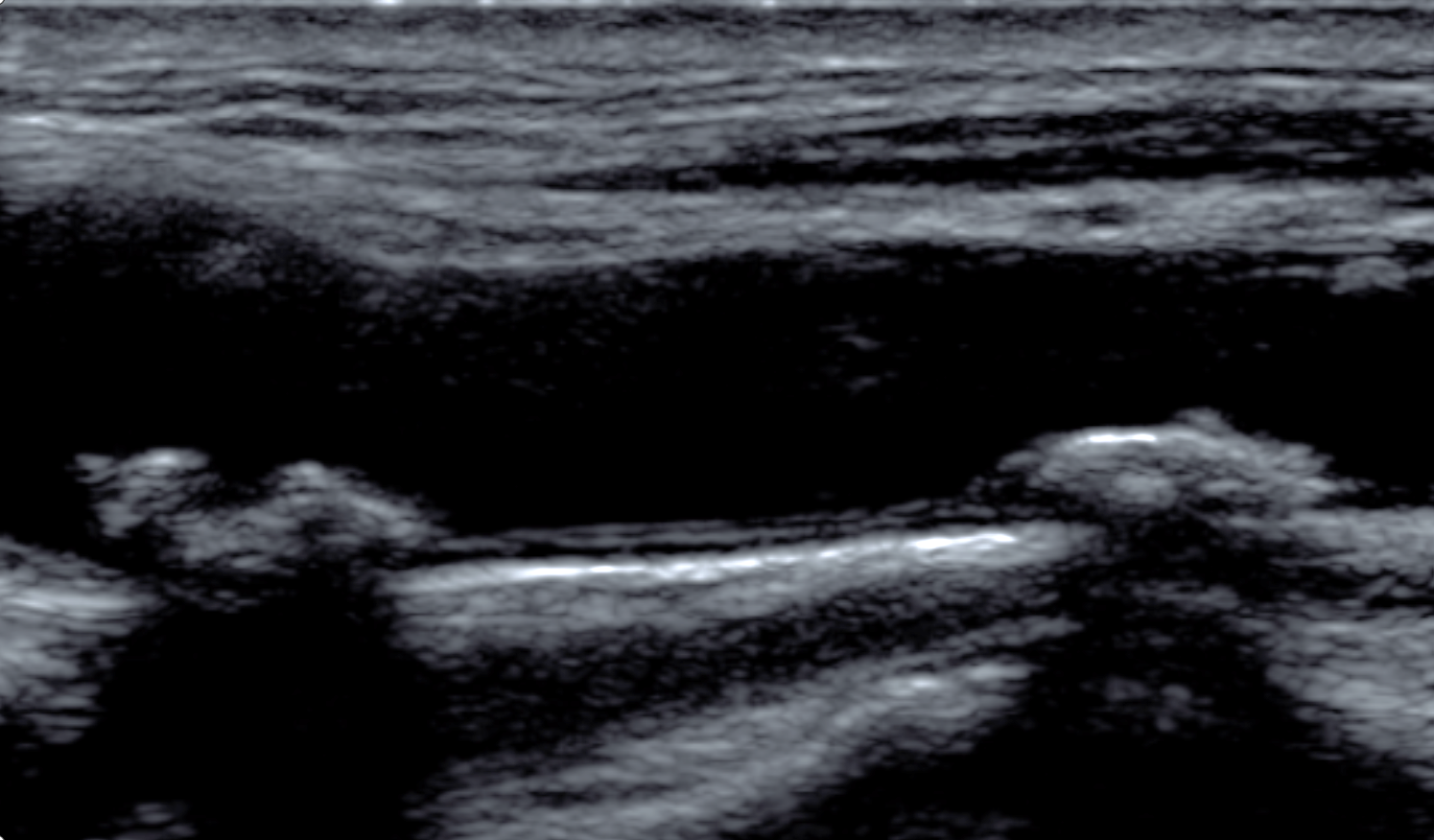 | (B) Plaque Ulceration |
| 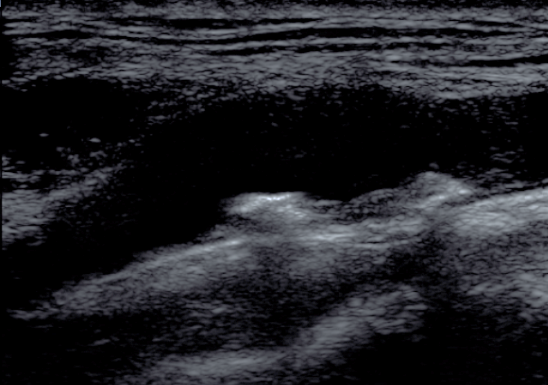 | (C) Irregular plaque |

| **Supplementary table 1.**  The difference of carotid plaque number and score according to ASCVD risk | | | |
| --- | --- | --- | --- |
|  |  | Number of plaques | cPS |
| FRS | Low | 1.52 ± 0.71 | 0.48 ± 1.40 |
|  | Intermediate | 1.67 ± 0.77 | 0.91 ± 1.86^a^ |
|  | High | 1.77 ± 0.77 | 1.18 ± 2.12^b^ |
| PCE | Low | 1.34 ± 0.63 | 0.39 ± 1.19^d^ |
|  | Borderline/Intermediate | 1.66 ± 0.76 | 0.82 ± 1.77 |
|  | High | 1.85 ± 0.78^c^ | 1.66 ± 2.43^d e^ |
| The number of carotid plaque and the cPS was increased as higher ASCVD risk. ^a^ *p*=0.002 versus low FRS risk; ^b^ *p*=0.012 versus low FRS risk; ^c^ p=0.002 versus low PCE risk; ^d^ *p*=0.001 versus borderline/intermediate PCE risk; ^e^ *p*<0.001 versus low PCE risk | | | |
| FRS, Framingham risk score; PCE, pooled cohort equation; cPS, carotid plaque score | | | |
